# A High-Capacity Ammonium Vanadate Cathode for Zinc-Ion Battery

**DOI:** 10.1007/s40820-020-0401-y

**Published:** 2020-03-04

**Authors:** Qifei Li, Xianhong Rui, Dong Chen, Yuezhan Feng, Ni Xiao, Liyong Gan, Qi Zhang, Yan Yu, Shaoming Huang

**Affiliations:** 1 Guangzhou Key Laboratory of Low-Dimensional Materials and Energy Storage Devices, School of Materials and Energy, Guangdong University of Technology, Guangzhou, 510006 People’s Republic of China; 2 Hefei National Laboratory for Physical Sciences at the Microscale, Department of Materials Science and Engineering, CAS Key Laboratory of Materials for Energy Conversion, University of Science and Technology of China, Hefei, 230026 People’s Republic of China; 3 Key Laboratory of Materials Processing and Mold (Zhengzhou University), Ministry of Education, Zhengzhou University, Zhengzhou, 450002 People’s Republic of China; 4 Aviation Fuel Research and Development Center, China National Aviation Fuel Group Limited, Beijing, 102603 People’s Republic of China; 5 Department Institute for Structure and Function and of Physics, Chongqing University, Chongqing, 400030 People’s Republic of China; 6 Dalian National Laboratory for Clean Energy (DNL), Chinese Academy of Sciences, Dalian, 116023 Liaoning People’s Republic of China; 7 State Key Laboratory of Fire Science, University of Science and Technology of China, Hefei, 230026 People’s Republic of China

**Keywords:** Zinc-ion battery, Ammonium vanadate, NH_4_V_4_O_10_

## Abstract

Given the advantages of being abundant in resources, environmental benign and highly safe, rechargeable zinc-ion batteries (ZIBs) enter the global spotlight for their potential utilization in large-scale energy storage. Despite their preliminary success, zinc-ion storage that is able to deliver capacity > 400 mAh g^−1^ remains a great challenge. Here, we demonstrate the viability of NH_4_V_4_O_10_ (NVO) as high-capacity cathode that breaks through the bottleneck of ZIBs in limited capacity. The first-principles calculations reveal that layered NVO is a good host to provide fast Zn^2+^ ions diffusion channel along its [010] direction in the interlayer space. On the other hand, to further enhance Zn^2+^ ion intercalation kinetics and long-term cycling stability, a three-dimensional (3D) flower-like architecture that is self-assembled by NVO nanobelts (3D-NVO) is rationally designed and fabricated through a microwave-assisted hydrothermal method. As a result, such 3D-NVO cathode possesses high capacity (485 mAh g^−1^) and superior long-term cycling performance (3000 times) at 10 A g^−1^ (~ 50 s to full discharge/charge). Additionally, based on the excellent 3D-NVO cathode, a quasi-solid-state ZIB with capacity of 378 mAh g^−1^ is developed.

**Electronic supplementary material:**

The online version of this article (10.1007/s40820-020-0401-y) contains supplementary material, which is available to authorized users.

## Highlights

- 3D flower-like architecture assembled by NH_4_V_4_O_10_ nanobelts (3D-NVO) was fabricated.
- The Zn^2+^ ion was intercalated into NVO cathode within the interlayer region (NH_4_V_4_O_10_ ↔ Zn_*x*_NH_4_V_4_O_10_).
- The 3D-NVO cathode could deliver a large reversible capacity of 485 mAh g^−1^ at a current density of 100 mA g^−1^ for zinc-ion battery.

**Electronic supplementary material:**

The online version of this article (10.1007/s40820-020-0401-y) contains supplementary material, which is available to authorized users.

## Graphical abstract

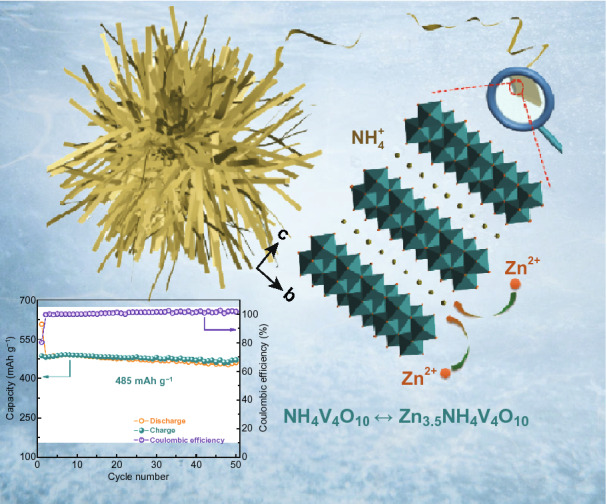

## Introduction

Battery technologies are the key to delivering significant advances in a wide range of industries, from portable electronics and electric vehicles to renewable power [1–5]. Given the looming concerns over the availability and safety hazards of lithium resources, rechargeable zinc-ion batteries (ZIBs) are relatively abundant in resources and environmental benign as compared to alkaline metals. To add in the easy manufacturing process, good safety characteristics and mature recycling process, ZIBs are the cost-effective solution for stationary applications in the long run as well [6–9]. Nevertheless, for most ZIBs, their specific capacity, cycling stability and rate capability are limited by cathode materials [10–13]. Developing cathode materials with outstanding electrochemical performance is imperative but yet remains a major challenge to be overcome.

The exploration focus of ZIB cathode materials remains on manganese-based oxides, such as *α*-MnO_2_ [14, 15], β-MnO_2_ [16], *γ*-MnO_2_ [17], *α*-Mn_2_O_3_ [18], Mn_3_O_4_ [19], and ZnMn_2_O_4_ [20, 21], which can deliver initial charge/discharge capacities up to 200–350 mAh g^−1^ under low rates. Nevertheless, their capacities decay drastically owing to the Mn dissolution via the disproportionation reaction upon repeated electrochemical cycling. Recent advances in reversible Zn^2+^ ion (de)intercalation in VO_2_ [22, 23], V_2_O_5_ [24–26], V_2_O_5_·*n*H_2_O [27, 28], Zn_0.25_V_2_O_5_·*n*H_2_O [29], H_11_Al_2_V_6_O_23.2_ [30], Ca_0.25_V_2_O_5_·*n*H_2_O [31], LiV_3_O_8_ [32], Na_0.33_V_2_O_5_ [33], Na_2_V_6_O_16_·3H_2_O [34], Mn_0.15_V_2_O_5_·*n*H_2_O [35], K_2_V_8_O_21_ [36], and VS_2_ [37] have motivated further exploration into vanadium-based cathodes for ZIBs. For example, a freestanding paper cathode of layered calcium vanadium oxide bronze demonstrates 340 mAh g^−1^ at a low current density [31] and layered Na_2_V_6_O_16_·3H_2_O as the host for Zn^2+^ ion conveys ~ 300 mAh g^−1^ (current rate: 180 mA g^−1^) and a high-rate performance [e.g., operating at 14.4 A g^−1^ (128 mAh g^−1^)] [34]. Despite current achievements, their reversible capacity is still far from being satisfactory (< 400 mAh g^−1^) owing to tardive Zn^2+^ diffusion.

The rich chemistry of ammonium vanadate (NH_4_V_4_O_10_, NVO) arising from the double layers of V_4_O_10_ and vanadium in high oxidation state makes it a great potential candidate for accommodating Zn^2+^ ions. In addition, the pillaring effect of NH_4_^+^ ions enlarges the interlayer spacing or “gallery” space (Fig. S1) [38], which promote Zn^2+^ ion diffusion along the tunnel (i.e., favorable electrochemical capacity and electrode kinetics). The successful reversible storage of Li^+^ ($r_{{{\text{Li}}^{ + } }}$ = 0.74 Å) [39–41], divalent Mg^2+^ ($r_{{{\text{Mg}}^{2 + } }}$ = 0.72 Å) [42], as well as larger-sized Na^+^ ($r_{{{\text{Na}}^{ + } }}$ = 1.02 Å) [43, 44], and Ca^2+^ ($r_{{{\text{Ca}}^{2 + } }}$ = 1.00 Å) [45] in NH_4_V_4_O_10_ as verified by theoretical calculations and experimental electrochemical measurements further predicts the feasibility of taking up Zn^2+^ cations with similar size ($r_{{{\text{Zn}}^{2 + } }}$ = 0.76 Å).

**Fig. 1:**
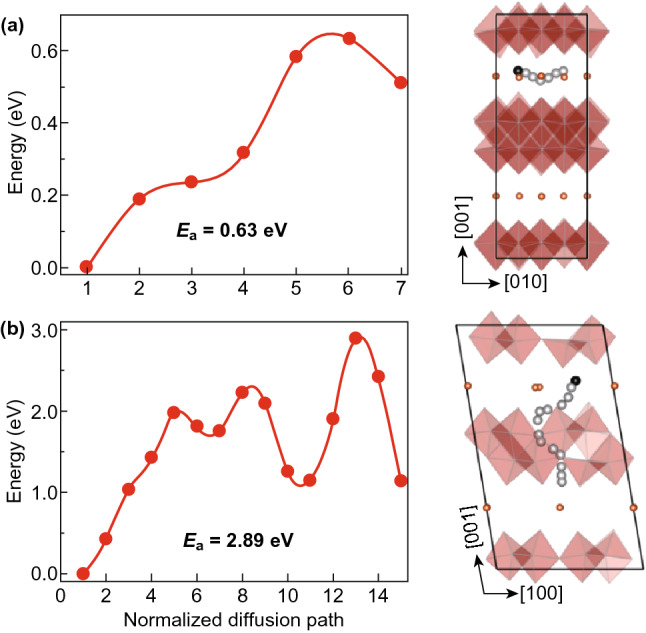
Calculated minimum energy paths of Zn^2+^ ion diffusion along **a** [010] and **b** [001] in monoclinic NH_4_V_4_O_10_. Orange and black balls represent NH_4_^+^ ions and the most energetically favorable pathway for Zn^2+^ ion intercalation, respectively. The VO layers are indicated by red polyhedrons. (Color figure online)

To prove the above prediction, we firstly performed the first-principles calculations to evaluate the Zn^2+^ ion intercalation behaviors in monoclinic NVO. Similar to the accommodation of Na^+^ ions [46], the intercalated Zn^2+^ ions energetically prefer the rest gallery space and sites. Using bulk Zn as the reference state, the binding energy was calculated to be − 3.18 eV, which is much stronger than that in layered V_2_O_5_ (− 2.06 eV) [47]. Figure S1 illustrates three possible migration paths: diffusion between two VO layers (i.e., along [010] and [100] directions) and that through VO layer (i.e., [001] direction). It is found that the migration of a Zn^2+^ ion directly along [100] direction seems impossible because the path is blocked by the NH_4_^+^ ions. Although the NH_4_^+^ ion that resides in the lattice spacing may be skirted between two NH_4_^+^ ions via diffusion, intercalation of Zn^2+^ ion at this site is extremely unstable as the initially placed Zn^2+^ ion would relax spontaneously to a nearby gallery site. Therefore, this possibility could be safely ruled out. The migration energy barrier, which reflects Zn^2+^ ion diffusivity, is determined by the maximum energy along each diffusion path. Figure 1 shows the calculated results of Zn^2+^ diffusion along [010] direction and through a VO layer in monoclinic NVO. Notably, lower energy barrier (0.63 eV) is demonstrated when Zn^2+^ ion diffuses along the [010] direction. In contrast, diffusion of a Zn^2+^ ion through a VO layer is also hindered as steric effect imposes a significantly large energy barrier (2.89 eV) for ion migration [48]. These results clearly suggest the feasibility of monoclinic NVO in providing fast Zn^2+^ ion intercalation along the [010] direction within the interlayer region.

**Fig. 2:**
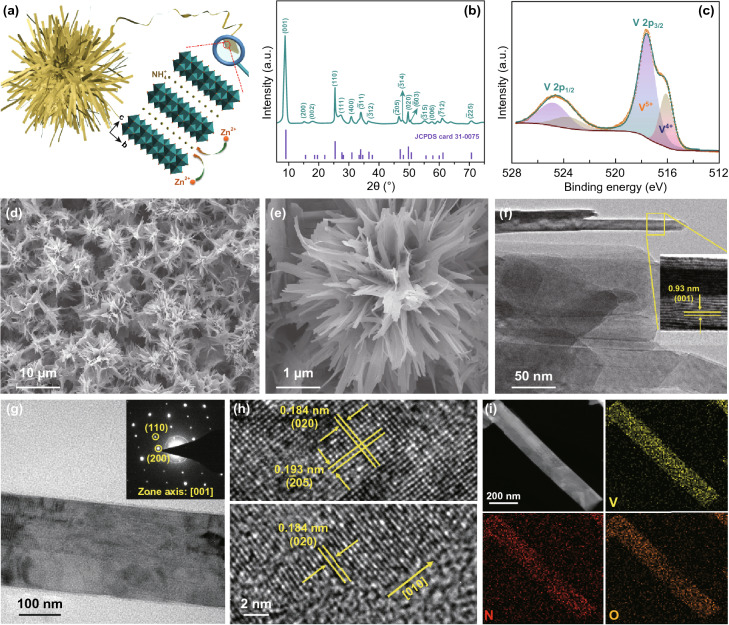
**a** Schematic illustration of the designed 3D-NVO architecture. Chemical and physical characterization of the 3D-NVO: **b** XRD pattern, **c** high-resolution XPS spectrum of the V 2p region, **d**, **e** SEM images, **f** TEM image (inset: HRTEM image of a lateral-lying nanobelt), **g** TEM image of an individual nanobelt (inset: the corresponding SAED pattern), **h** HRTEM images, and **i** STEM and the corresponding elemental mapping images

The electrochemical performance of ZIBs, especially at high charging/discharging rates, is mainly determined by the Zn^2+^ ion solid-state diffusion process (i.e., rate-determining step) in the electrode materials. Thus, it is highly desirable to minimize the dimensions of NVO active materials in order to accelerate the Zn^2+^ ion kinetics. Thus, we attempt to synthesize one-dimensional (1D) NVO nanobelts to achieve this goal. Meanwhile, to avoid the agglomeration of NVO nanobelts, NVO nanobelts are designed to self-assemble into three-dimensional (3D) flower-like architecture (abbreviated as 3D-NVO, Fig. 2a), which can maintain structural integrity during repeated Zn^2+^ (de)intercalation. Although there are some reports on the fabrication of 1D NVO [43, 49, 50], engineering them into 3D-NVO presents a great challenge but might bring a significance to the development of electrode materials for ZIBs and quasi-solid-state ZIBs.

## Experimental

### Synthesis of 3D-NVO

In a typical synthesis, 2 mmol ammonium metavanadate (NH_4_VO_3_) and 2 mmol oxalic acid (H_2_C_2_O_4_) were added into deionized (DI) water (30 mL) under magnetic stirring, creating a kelly green solution. Then, it was put into an autoclave (50 mL) and placed in a microwave oven heated under 180 °C for 30 min. Afterward, the product was washed with DI water and ethanol and then frozen under liquid nitrogen to subject a vacuum drying process for about 48 h.

### Calculation Methods

Vienna Ab-Initio Simulation Package was adopted to conduct first-principles calculations. The spin-polarized Perdew–Burke–Ernzerhof generalized gradient approximation was used for the exchange–correlation functional [51–53]. Van der Waals correction was included using the Grimme scheme (D2) for simulations of monoclinic NVO (space group: C2/m) [54]. The lattice parameters of NVO were calculated to be *a* = 11.79 Å, *b* = 3.68 Å, *c* = 9.93 Å and *β* = 99.49°, consistent well with experimental values. A (1 × 2 × 2) supercell involving 152 atoms (N_8_H_32_V_32_O_80_) was constructed to simulate diffusion behavior of zinc ions in NVO. It was relaxed with force convergence criteria of 0.01 eV Å^−1^. Moreover, 500 eV cutoff energy and Γ-centered 3 × 5 × 2 *k*-mesh were used. The climbing image nudged elastic band method was adopted to achieve the pathway with the minimum energy for Zn ion diffusion [55]. Because of the strong electronic correlations in the localized *d* orbitals of V ions, an effective parameter of U–J = 4.0 eV was applied for an on-site Coulomb interaction, which was believed to be able to provide more appropriate description of electronic properties in vanadium oxides [56].

### Materials Characterization

The X-ray diffraction (XRD) patterns were acquired from an advanced X-ray diffractometer with Cu *K*_*α*_ radiation (Bruker D8). A field-emission scanning electron microscopy (SEM) system (Hitachi, Model SU8220) was used to investigate the morphology of the products. To further determine their microstructures, transmission electron microscopy (TEM) characterization (FEI, Model Talos F200S) operating at 200 kV was carried out. The X-ray photoelectron spectroscopy (XPS) was measured on ESCALAB 250Xi (Thermo Fisher).

### Electrochemical Measurements

The electrochemical performance of the 3D-NVO materials was tested by assembling them into coin-type cells in air. Typically, 80 wt% 3D-NVO was mixed with 10 wt% carbon nanotubes and 10 wt% poly(vinylidenefluoride) (PVDF) in the solvent of *N*-methylpyrrolidone (NMP). The as-formed slurry was then pasted onto the titanium foils (diameter: 1 μm) to achieve the cathode part, which was later coupled with zinc foils anode and 1 M Zn(ClO_4_)_2_ in propylene carbonate (PC) as the electrolyte, for complete coin-cell configuration. In addition, quasi-solid-state ZIBs were assembled using the as-prepared 3D-NVO cathode, zinc foil anode, and the electrolyte of solid polymer membranes. The solid membranes were prepared as follows: (1) 0.264 g Zn(ClO_4_)_2_ and 0.75 g PVDF were initially dissolved in 10 mL N, N-dimethylformamide (DMF) under magnetic stirring, (2) adding 1 mL of zinc bis(trifluoromethanesulfonyl)imide (Zn(TFSI)_2_, 1 M) in 1-ethyl-3-methylimidazolium bis(trifluoromethylsulfonyl)imide (EMIMTFSI), and (3) the mixed solution was poured into the mold and dried at vacuum oven for 8 h under 80 °C. The tests were carried out on a Neware battery system, and the discharging/charging profiles were performed within 0.3–1.5 V (vs. Zn^2+^/Zn). Cyclic voltammetry was performed with a IVIUM electrochemical workstation.

## Results and Discussion

In this paper, 3D-NVO cathode materials were successfully synthesized by reacting ammonium metavanadate (NH_4_VO_3_) with oxalic acid (H_2_C_2_O_4_) in aqueous solution through a one-pot microwave-assisted hydrothermal method. Figure 2b shows the XRD pattern of 3D-NVO sample. All the diffraction peaks are readily assigned to a pure monoclinic structure of NH_4_V_4_O_10_ (JCPDS Card No. 31-0075), agreeing well with the literature [49, 57]. The intensity of (001) peak is extremely high, which illustrates the preferential exposure of (001) surface facets. Its elemental composition and chemical states were further identified by X-ray photoelectron spectroscopy (XPS). V, N, and O elements are presented, and two peaks at approximately 401.2 and 530.2 eV are the characteristic binding energies of N and O, respectively (Fig. S2). Meanwhile, high-resolution XPS spectrum of V 2p in Fig. 2c is split into the overlapped V^5+^ (2p_3/2_: 517.4 eV) and V^4+^ (2p_3/2_: 516.4 eV) peaks, displaying the average vanadium oxidation state of + 4.74, proving the sectional reduction of pentavalent vanadium by oxalic acid [58].

SEM and TEM were employed to examine the morphology and microstructure of the as-prepared 3D-NVO. The low-magnification SEM image in Fig. 2d reveals the uniform 3D microflower-like morphology of the 3D-NVO, and each of them shows a diameter in the range of 4–10 μm. Magnified SEM image (Fig. 2e) and TEM examination (Figs. 2f and S3) indicate that hierarchical microflowers are constructed by numerous NVO nanobelts with a length of 2–5 μm and a width of 100–200 nm. Moreover, a lateral-lying nanobelt found in Fig. 2f displays the side view of the nanobelt and its thickness is estimated to be around 20 nm. The high-resolution TEM (HRTEM) observation further demonstrates that the interplanar spacing of the layered NVO along *c*-axis direction is as large as 0.93 nm (inset of Fig. 2f). The selected-area electron diffraction (SAED) pattern of an individual NVO nanobelt (Fig. 2g) unveils single-crystalline character by showing well-defined diffraction spots (zone axis: [001]). HRTEM image glimpsed from the middle of a nanobelt (Fig. 2h, upper panel) exhibits two sets of lattices with *d*-spacings of 0.184 and 0.193 nm, matching well with the (020) and (− 205) planes of monoclinic NVO, respectively. In addition, from the HRTEM observation on the edge of a nanobelt (Fig. 2h, bottom panel), we can see the lattice fringes (*d*-spacing of 0.184 nm for (020) planes) perpendicular to the length of the nanobelt, illustrating the preferential orientation growth along the [010] direction. Furthermore, Fig. 2i depicts the scanning TEM (STEM) and the corresponding elemental mapping of a NVO nanobelt, indicating uniform distribution of V, N, and O elements.

The in situ self-assembly of NVO nanobelts into 3D microflowers is driven by microwave irradiation heating (MIH), which is a very fast (30 min). To announce the importance of MIH, conventional autoclave hydrothermal reactions (C-HT) at different times were carried out, while other experimental parameters are kept the same as those of MIH. It was found that, as shown in Fig. S4, only NVO nanobelts without 3D assemblies could be produced in C-HT even after prolonged reaction time of 2, 6, and 12 h (almost no sample was presented after 30 min of reaction). Furthermore, under the method of C-HT, NVO nanobelt morphology was also widely observed in the literature using similar reactants, reaction temperatures, and time [41, 42, 46, 49, 50]. Hence, the role of MIH appears to be critical for a successful growth of the 3D-NVO, which can directly interact with reactants by producing more homogeneous heat distribution at molecular level throughout the entire solution as compared to C-HT (Fig. S5), hence reducing reaction time and enhancing reaction kinetics [59].

**Fig. 3:**
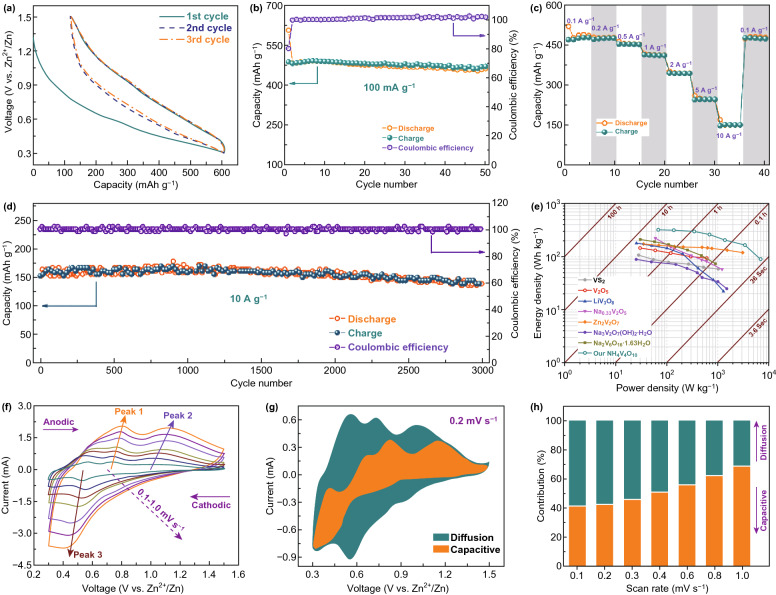
Electrochemical characterization of the 3D-NVO cathode. **a** Galvanostatic discharge and charge profiles for the first three cycles at 100 mA g^−1^. **b** Cycling performance at 100 mA g^−1^. **c** Rate capability. **d** Long-term cycling performance at a high rate of 10 A g^−1^. **e** Ragone plots of our 3D-NVO cathode, compared with some advanced vanadium-based cathodes for ZIBs. **f** CV curves at scan rates from 0.1 to 1.0 mV s^−1^. **g** Evaluation of capacitive contribution to the total charge storage (e.g., 0.2 mV s^−1^). **h** Contribution ratio of the capacitive-controlled and diffusion-controlled capacities at various scan rates

Electrochemical performance of the 3D-NVO cathode was evaluated in coin-type cells. Figure 3a shows its voltage-capacity profiles at a low rate of 100 mA g^−1^ for the first three cycles. Zn^2+^ intercalation (discharge) and deintercalation (charge) are represented by the S-shaped sloping curves, which correspond to solid solution processes. After the first discharge, a capacity of 607 mAh g^−1^ is achieved, and followed by a charge capacity of 487 mAh g^−1^, denoting a Coulombic efficiency (CE) of 80%. As shown in Fig. 3b, the capacity illustrates an outstanding cycling performance with almost no capacity fading during the subsequent cycles, and the CE is close to 100% from the 2nd cycle onward. Remarkably, the sustained reversible capacity (485 mAh g^−1^, i.e., uptake/release of 3.5 Zn per unit formula of NVO) is extraordinarily high, which has not yet been achieved for the reported ZIB cathodes (Table S1). Good high-rate performance is essential to build fast-charging ZIBs. As demonstrated in Fig. 3c, the cell was run between 0.1 and 10 A g^−1^. Capacities of 486, 475, 453, 411, 343, and 246 mAh g^−1^ are displayed at current densities of 0.1, 0.2, 0.5, 1.0, 2.0, and 5.0 A g^−1^, respectively, and the sloping discharge/charge profiles are also well retained at various rates (Fig. S6). Moreover, a capacity of 142 mAh g^−1^ is still delivered even at 10 A g^−1^ (approximately 50 s to full discharge/charge). Particularly, even after rapid charging/discharging, the 3D-NVO can still recover to high capacity of 478 mAh g^−1^ when the rate is returned to 0.1 A g^−1^. Furthermore, the stability of this cathode at 10 A g^−1^ is superior, showing almost no capacity loss after 3000 cycles (Fig. 3d) and unchanged discharge/charge profiles (Fig. S7). In contrast, the cathode of NVO nanobelts obtained from the traditional hydrothermal method (Fig. S4c, d) demonstrates a fast capacity decaying (only 58% capacity retention after 300 cycles) at 10 A g^−1^ (Fig. S8), confirming the superiority of the 3D-NVO cathode.

The 3D-NVO is placed in a Ragone plot (energy density vs. power density) based on working potential, specific capacity, and current density for performance evaluation. Encouragingly, as displayed in Fig. 3e, our 3D-NVO exhibits a very high energy density of 321 Wh kg^−1^ at a specific power of 69 W kg^−1^. Furthermore, an outstanding power density of 6.9 kW kg^−1^ (corresponding energy density: 90 Wh kg^−1^) can also be obtained. These results pass significantly beyond most advanced ZIB cathodes such as VS_2_ [37], V_2_O_5_ [25], LiV_3_O_8_ [32], Na_0.33_V_2_O_5_ [60], Zn_2_V_2_O_7_ [61], Na_3_V_2_O_7_(OH)_2_·2H_2_O [62], and Na_2_V_6_O_16_·1.63H_2_O [63], evidencing the superior ZIBs performance of our 3D-NVO materials.

To understand the electrochemical Zn^2+^ ion storage kinetics of 3D-NVO cathode, cyclic voltammetry (CV) was performed under scan rates of 0.1–1.0 mV s^−1^ (Fig. 3f). The redox peaks are gradually broadened and slightly shifted with increasing scan rate, but the CV contour is maintained. Their current (*i*), in principle, is obedient to a power-law relationship with the scan rate (*ν*) via the equation: *i* = a*ν*^*b*^. When the *b* value is 0.5, it represents a diffusion-controlled process, whereas *b* = 1.0 stands for a capacitive process. Accordingly, the data in Fig. S9 reveal a combination of Zn^2+^ ion intercalation and capacitive reactions in the 3D-NVO cathode. (*b* values range from 0.78 to 0.86 for the marked cathodic and anodic peaks in Fig. 3f.) Again, based on the equation of *i*(V) = *k*_1_*ν* (capacitive effect) + *k*_2_*ν*^1/2^ (diffusion effect), their proportion in the total stored charge can be quantified (e.g., the orange area in Fig. 3g corresponds to the capacitive contribution). Figure 3h illustrates the contributions from the two different charge storage mechanisms, and we find that the capacitive contribution increases from 40.8 to 68.4% with an increasing scan rate (0.1–1.0 mV s^−1^), which is primary factor that enables fast reaction kinetics and superior high-rate performance.

**Fig. 4:**
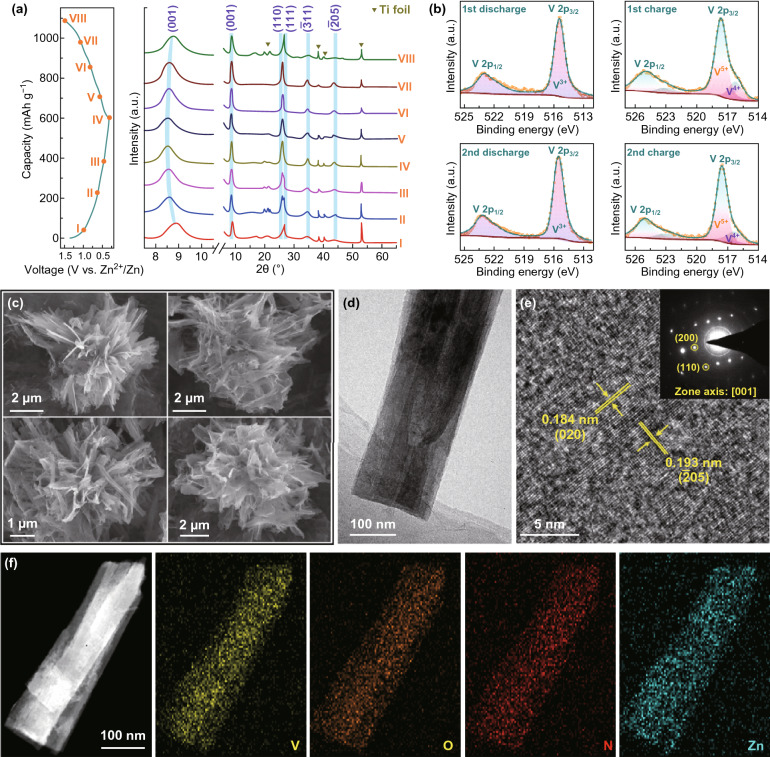
Postmortem analysis of the 3D-NVO cathode. **a** Ex situ XRD patterns during the first cycle. **b** High-resolution XPS spectra of the V 2p region at fully discharged and charged states during the first two cycles. **c** SEM, **d** TEM, **e** HRTEM (inset: SAED pattern), and **f** STEM and the corresponding elemental mapping images of the cathode after 50 cycles at 100 mA g^−1^

To investigate the storage mechanism of Zn^2+^ ion in the 3D-NVO cathode, ex situ XRD experiments at different charge–discharge states were performed to examine its structural evolution (Fig. 4a). Clearly, there is no significant change in the XRD patterns under various states of discharge/charge and the characteristic peaks of NH_4_V_4_O_10_ located at around 9.0° (001), 25.5° (110), 27.7° (111), 34.0° (− 311), and 44.6° (− 205) are retained without detecting any new diffraction peaks, which is indicative of the well-preserved lamellar structure. In addition, with a careful observation, it is found that the (001) peak is slightly shift toward lower angles during the discharge process, confirming the Zn^2+^ ion intercalation reaction mechanism with an expansion of the NH_4_V_4_O_10_ interlayer spacing. When Zn^2+^ ions are completely released, this peak can be recovered, demonstrating the reversible behavior of the NH_4_V_4_O_10_ lattice layer. The varying of vanadium chemical states upon Zn^2+^ ion (de)intercalation was further evaluated by ex situ XPS analysis (Fig. 4b). At the end of the 1^st^ discharge state, the V 2p peaks can be fitted to V^3+^ (2p_3/2_: 515.5 eV) specie, which is consistent with the fully discharged product (Zn_3.5_NH_4_V_4_O_10_) derived from the galvanic curve. (485 mAh g^−1^ corresponds to an uptake of 3.5 Zn^2+^ ion per formula unit.) Upon subsequent charging process, the V 2p spectrum almost fully recovers to its pristine state (V^4.74+^) in Fig. 2c. The XPS V 2p spectra under the 2^nd^ cycle further evidence the reversible transition between V^4.74+^ and V^3+^ via the reaction of NH_4_V_4_O_10_ + 3.5Zn^2+^ + 7.0e^−^ ↔ Zn_3.5_NH_4_V_4_O_10_. Furthermore, SEM images of the cycled 3D-NVO cathode reveal that the pristine 3D microflower-like structure is well maintained (Fig. 4c), and the surface of NVO nanobelt is very smooth without experiencing severe pulverization (Fig. 4d). The HRTEM image with clear lattice fringes and the spotted SAED pattern suggests its highly crystalline nature is preserved (Fig. 4e). The scanning TEM (STEM) image and the corresponding elemental mapping illustrate a homogeneous distribution of V, O, N, and Zn in the full discharged NVO nanobelt (Fig. 4f). Additionally, the crystal structure of NH_4_V_4_O_10_ is still retained after 100 cycles at 10 A g^−1^ (Fig. S10). On the basis of these ex situ XRD, XPS, SEM, and TEM results, we believe that the crystal structure and microstructure of our 3D-NVO cathode are highly stable and reversible upon Zn^2+^ ion intercalation/deintercalation, confirming superior electrochemical performance.

**Fig. 5:**
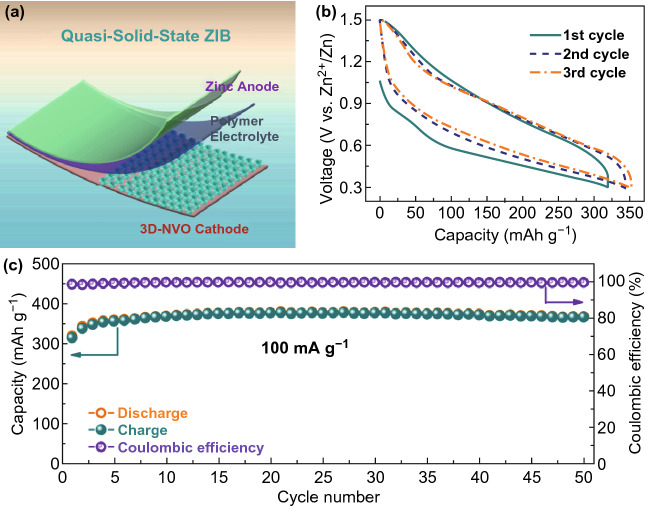
**a** Schematic illustration of the configuration of the quasi-solid-state ZIB composed of the 3D-NVO cathode. **b** The charge/discharge profiles for initial three cycles and **c** cycling performance at 100 mA g^−1^

Solid-state (quasi-solid-state) batteries with high safety are appealing great attention for energy storage applications [64–67]. Hence, we assembled a quasi-solid-state ZIB based on 3D-NVO cathode, zinc anode, and the electrolyte and separator of poly(vinylidenefluoride)-Zn(ClO_4_)_2_-based polymer membrane (Fig. 5a). Figure 5b shows the charge–discharge curves at 100 mA g^−1^. Obviously, the profiles are the same as those of liquid ZIB in Fig. 3a, sharing the above-mentioned Zn^2+^ (de)intercalation mechanism. During the first cycle, specific capacities of 320 (discharge) and 315 mAh g^−1^ (charge) are achieved with a CE of 98%. Remarkably, in the following cycles, the specific capacity is slightly increased (corresponding to the activation process) and stabilized at 378 mAh g^−1^ with CE of about 100% after 50 cycles (Fig. 5c), which is much higher than state-of-the-art quasi-solid-state ZIBs (e.g., ~ 200 mAh g^−1^ for zinc orthovanadate array//Zn array [68], and ~ 300 mAh g^−1^ for V_5_O_12_·6H_2_O nanobelts//Zn foil [69], etc.), making it promising for solid-state energy storage systems.

## Conclusions

In summary, 3D-NVO cathode was created with high capacity for ZIBs. Firstly, the first-principles calculations were carried out to confirm feasibility of Zn^2+^ intercalation into monoclinic NVO, in which the intercalates tend to accommodate in the interlayer region of NVO along the [010] direction. Subsequently, to enhance the Zn^2+^ ion diffusion kinetics and maintain the structural integrity of the electrode during long-term cycling process, a 3D flower-like architecture assembled by NVO nanobelts was designed and fabricated using a microwave-assisted hydrothermal method. In ZIB application, this 3D-NVO cathode can bring a large reversible capacity of 485 mAh g^−1^ (corresponds to energy density of 321 Wh kg^−1^) under current density of 0.1 A g^−1^. Additionally, superior long-term (3000 times) high-rate cycling performance is demonstrated (i.e., power density: 6.9 kW kg^−1^). Postmortem investigation of cycled 3D-NVO further identifies that 3.5 Zn^2+^ ion is taken up upon intercalation through the following reaction: NH_4_V_4_O_10_ ↔ Zn_3.5_NH_4_V_4_O_10_ without affecting the crystallinity and microstructure of the pristine 3D-NVO. Finally, a high-capacity (378 mAh g^−1^) quasi-solid-state ZIB composed of the 3D-NVO cathode is developed.

## Electronic supplementary material

Below is the link to the electronic supplementary material.

Supplementary material 1 (PDF 866 kb)
