# Supplementary material for: A High-Capacity Ammonium Vanadate Cathode for Zinc-Ion Battery
Source: Nanomicro Lett. 2020 Mar 4;12:67. doi: 10.1007/s40820-020-0401-y (PMC7770878; doi:10.1007/s40820-020-0401-y)
Supplement: Supplementary file 1 — Supplementary material 1 (PDF 866 kb) [file 40820_2020_401_MOESM1_ESM.pdf]

Supporting Information for

A High Capacity Ammonium Vanadate Cathode for Zinc-ion Battery

Qifei Li[1], Xianhong Rui[1, 2, *], Dong Chen[1], Yuezhan Feng[3], Ni Xiao[4], Liyong Gan[5], Qi Zhang[1], Yan Yu[2, 6, 7, *], Shaoming Huang[1, *]

[1]Guangzhou Key Laboratory of Low-Dimensional Materials and Energy Storage Devices, School of Materials and Energy, Guangdong University of Technology, Guangzhou 510006, People's Republic of China

[2]Hefei National Laboratory for Physical Sciences at the Microscale, Department of Materials Science and Engineering, CAS Key Laboratory of Materials for Energy Conversion, University of Science and Technology of China, Hefei 230026, People's Republic of China

[3]Key Laboratory of Materials Processing and Mold (Zhengzhou University), Ministry of Education, Zhengzhou University, Zhengzhou 450002, People's Republic of China

[4]Aviation Fuel Research & Development Center, China National Aviation Fuel Group Limited, Beijing 102603, People's Republic of China

[5]Institute for Structure and Function and Department of Physics, Chongqing University, Chongqing 400030, People's Republic of China

[6]Dalian National Laboratory for Clean Energy (DNL), Chinese Academy of Sciences, Dalian, Liaoning 116023, People's Republic of China

[7]State Key Laboratory of Fire Science, University of Science and Technology of China, Hefei 230026, People's Republic of China

*Corresponding authors. E-mail: xhrui@gdut.edu.cn (X.H. Rui); yanyumse@ustc.edu.cn (Y. Yu); smhuang@gdut.edu.cn (S.M. Huang)

Supplementary Figures and Table

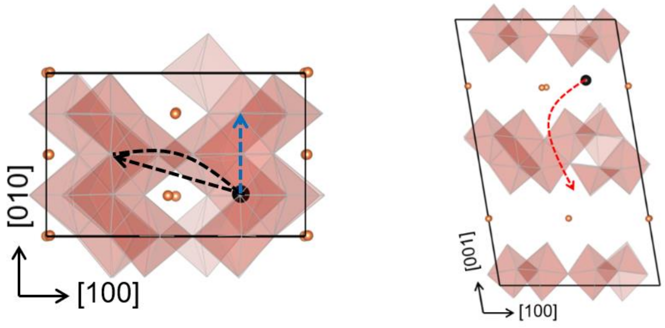

Fig. S1 Schematics of possible diffusion pathways for Zinc ion diffusion in monoclinic $NH_4V_4O_{10}$ viewed along the [100] (black arrows), [010] (blue arrow) and [001] (red arrow) direction. Orange and black balls indicate NH_4^+ ions and the most energetically

favorable location for Zn^{2+} intercalation, respectively. The VO layers are represented by red polyhedrons.

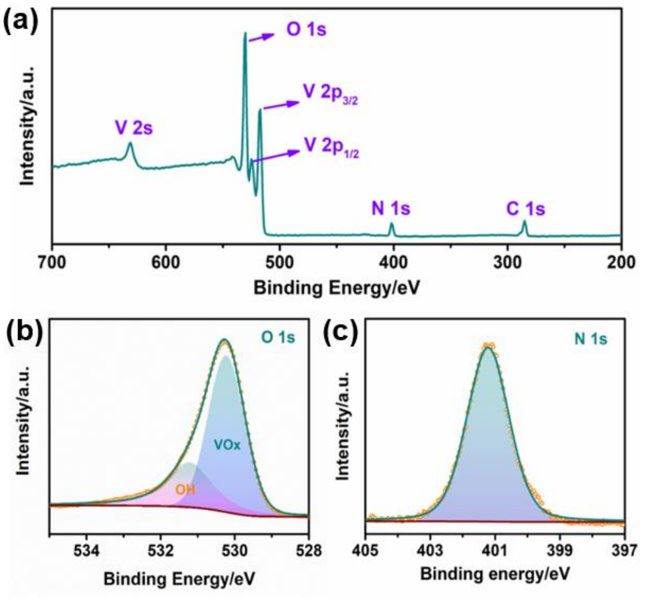

Fig. S2 The full XPS spectrum **(a)**, and high-resolution XPS spectra of O **(b)** and N **(c)** species.

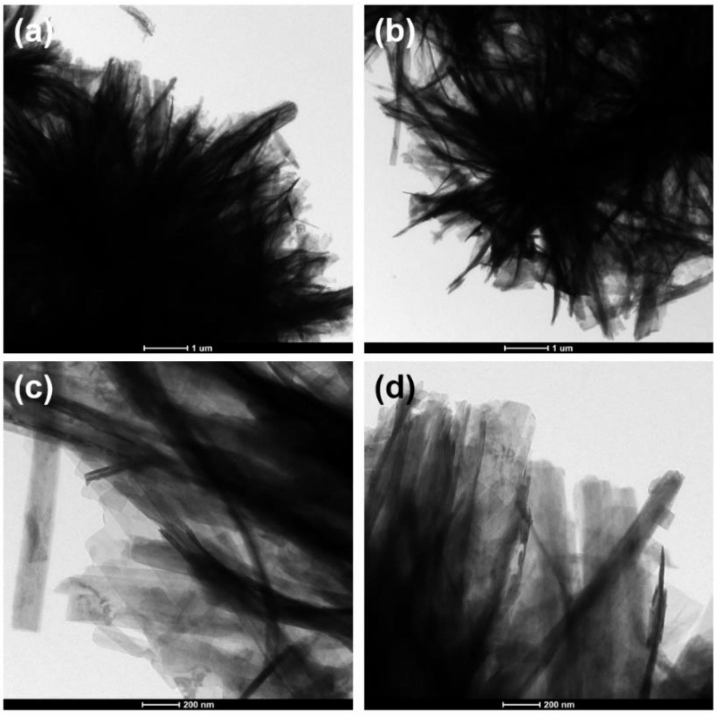

Fig. S3 TEM images of the 3D-NVO sample at different magnifications

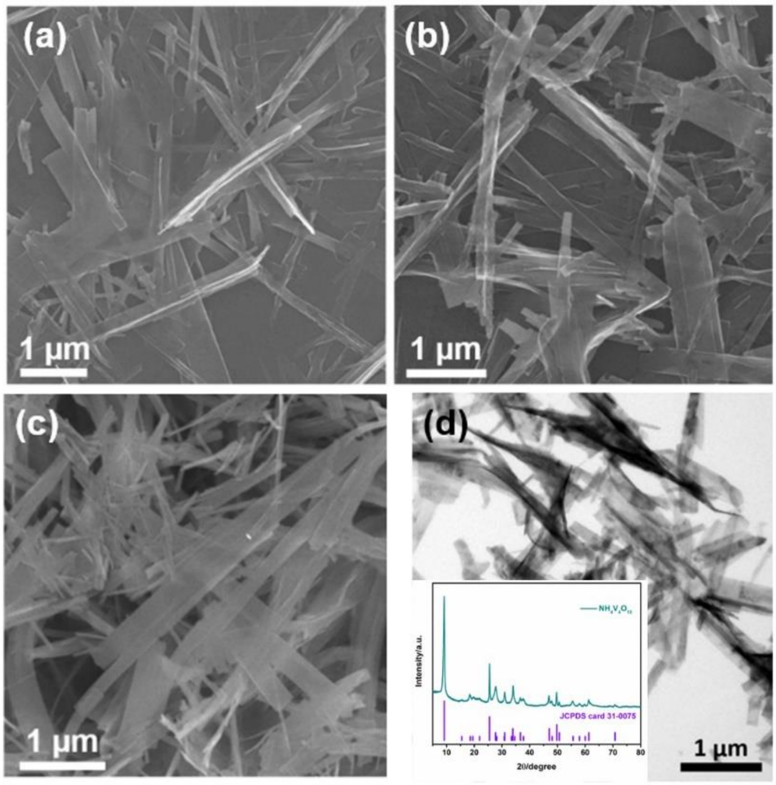

Fig. S4 (a-c) SEM and **(d)** TEM images of the sample prepared by conventional autoclave hydrothermal method at reaction time of 2 h **(a)**, 6 h **(b)** and 12 h **(c and d)**. Inset in (d) is the corresponding XRD pattern obtained at 12 h, which can be assigned to a pure monoclinic structure of $NH_4V_4O_{10}$ (JCPDS Card No. 31-0075).

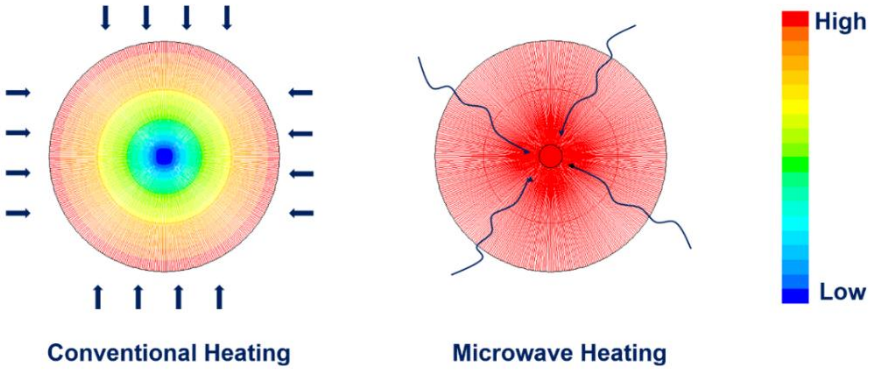

Fig. S5 Schematic illustration of the difference between microwave and conventional heating. In conventional heating system, there is a temperature gradient from the outside to the inner core.

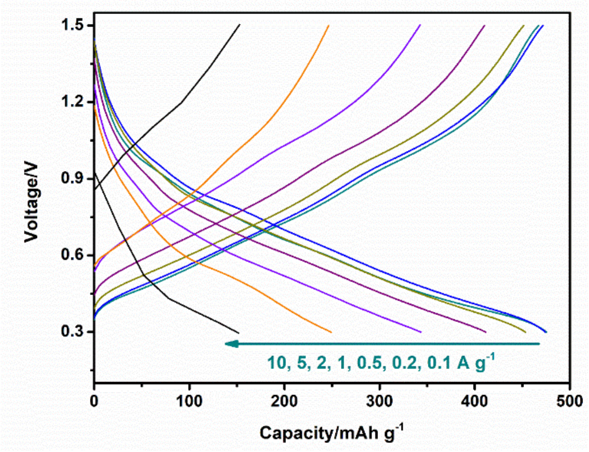

Fig. S6 Galvanostatic discharge and charge profiles of the 3D-NVO cathode at different current densities

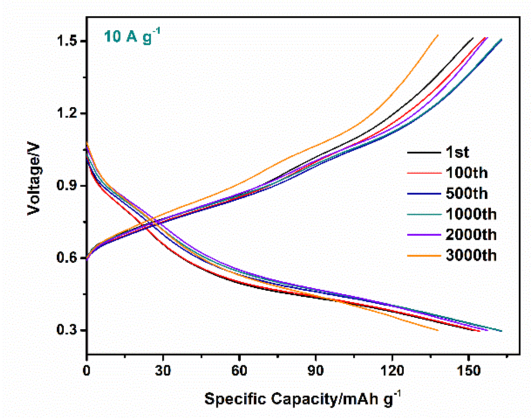

Fig. S7 Galvanostatic discharge and charge profiles of the 3D-NVO cathode during different cycles at 10 A g^{-1}

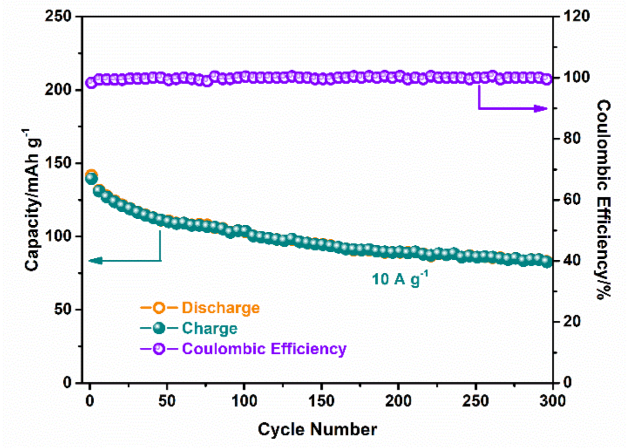

Fig. S8 Cycling performance of the NVO nanobelts obtained from the traditional hydrothermal method at 10 A g^{-1}

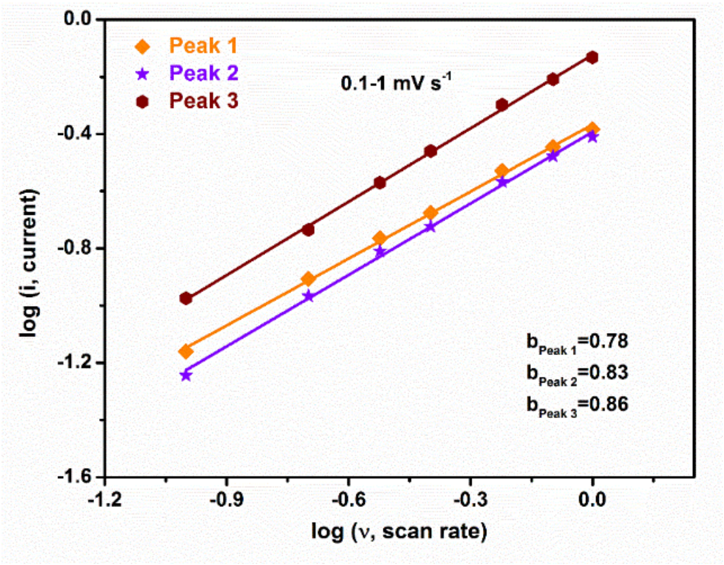

Fig. S9 Plots of log(i) vs. log(v) based on anodic and cathodic peaks in Fig. 3f

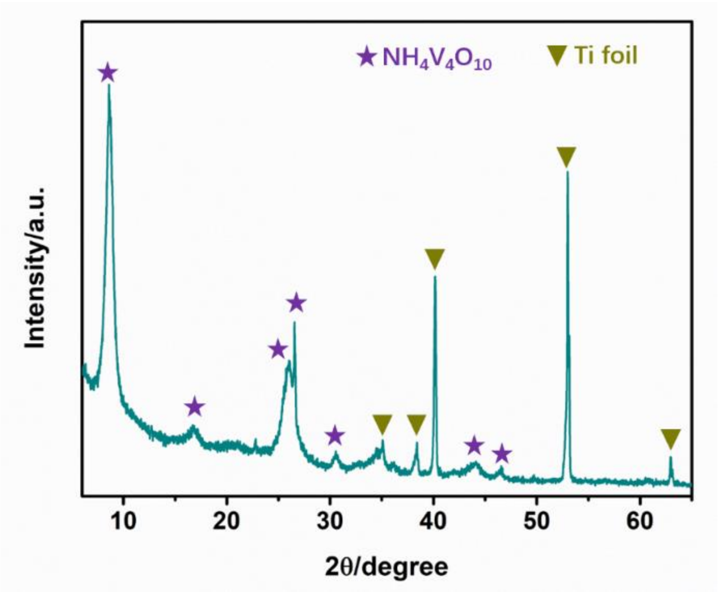

Fig. S10 The XRD pattern of the 3D-NVO cathode after 100 cycles at 10 A g^{-1}

Table S1 A comparison of the reversible capacity of our 3D-$NH_4V_4O_{10}$ cathode to previously reported superior ZIB cathodes

Cathode materials	Reversible capacity [mAh g^{-1}]	Rate [mA g^{-1}]	Number of cycles	Capacity retention
**This work (3D-$NH_4V_4O_{10}$)**	**486**	**100**	**50**	**98%**
α-MnO_2 [S1]	233	83	50	63%
δ-MnO_2 [S2]	225	83	100	50%
Mn_3O_4 [S3]	195	200	70	67%
$ZnMn_2O_4$ [S4]	106	100	300	59%
VO_2 [S5]	357	100	50	99%
VS_2 [S6]	138	200	200	80%
V_2O_5 [S7]	215	100	160	95%
$V_2O_5 \cdot nH_2O$ [S8]	196	14.4	120	87%
$H_2V_3O_8$@Graphene [S9]	360	300	150	93%
LiV_3O_8 [S10]	185	133	65	78%
$Na_{0.33}V_2O_5$ [S11]	276	200	100	91%
$Zn_2V_2O_7$ [S12]	210	300	200	94%
$Zn_3V_2O_7(OH)_2 \cdot 2H_2O$ [S13]	149	200	300	68%
$Na_2V_6O_{16} \cdot 1.63H_2O$ [S14]	296	100	100	78%
$Na_{1.1}V_3O_{7.9}$@rGO [S15]	200	50	30	87%
$V_{1-x}Al_xO_{1.52}(OH)_{0.77}$ [S16]	156	15	50	68%
$Na_3V_2(PO_4)_3$@C [S17]	97	50	100	74%

Supplementary References

[S1] M.H. Alfaruqi, J. Gim, S. Kim, J. Song, J. Jo, S. Kim, V. Mathew, J. Kim, Enhanced reversible divalent zinc storage in a structurally stable alpha-MnO_2 nanorod electrode. J. Power Sources **288,** 320-327 (2015). https://doi.org/10.1016/j.jpowsour.2015.04.140

[S2] M.H. Alfaruqi, J. Gim, S. Kim, J. Song, P. Duong Tung et al., A layered delta-MnO_2 nanoflake cathode with high zinc-storage capacities for eco-friendly battery applications. Electrochem. Commun. **60,** 121-125 (2015). https://doi.org/10.1016/j.elecom.2015.08.019

[S3] J. Hao, J. Mou, J. Zhang, L. Dong, W. Liu, C. Xu, F. Kang, Electrochemically induced spinel-layered phase transition of Mn_3O_4 in high performance neutral aqueous rechargeable zinc battery. Electrochim. Acta **259,** 170-178 (2018). https://doi.org/10.1016/j.electacta.2017.10.166

[S4] X. Wu, Y. Xiang, Q. Peng, X. Wu, Y. Li et al., Green-low-cost rechargeable

aqueous zinc-ion batteries using hollow porous spinel $ZnMn_2O_4$ as the cathode material. J. Mater. Chem. A **5,** 17990-17997 (2017). https://doi.org/10.1039/C7TA00100B

[S5] J. Ding, Z. Du, L. Gu, B. Li, L. Wang, S. Wang, Y. Gong, S. Yang, Ultrafast Zn^{2+} intercalation and deintercalation in vanadium dioxide. Adv. Mater. **30,** 1800762 (2018). https://doi.org/10.1002/adma.201800762

[S6] P. He, M. Yan, G. Zhang, R. Sun, L. Chen, Q. An, L. Mai, Layered VS_2 nanosheet-based aqueous zn ion battery cathode. Adv. Energy Mater. **7,** 1601920 (2017). https://doi.org/10.1002/aenm.201601920

[S7] P. Hu, M. Yan, T. Zhu, X. Wang, X. Wei et al., Zn/V_2O_5 aqueous hybrid-ion battery with high voltage platform and long cycle life. ACS Appl. Mater. Interfaces **9,** 42717-42722 (2017). https://doi.org/10.1021/acsami.7b13110

[S8] P. Senguttuvan, S.D. Han, S. Kim, A.L. Lipson, S. Tepavcevic et al., A high power rechargeable nonaqueous multivalent Zn/V_2O_5 battery. Adv. Energy Mater. **6,** 1600826 (2016). https://doi.org/10.1002/aenm.201600826

[S9] Q. Pang, C. Sun, Y. Yu, K. Zhao, Z. Zhang et al., $H_2V_3O_8$ nanowire/graphene electrodes for aqueous rechargeable zinc ion batteries with high rate capability and large capacity. Adv. Energy Mater. **8,** 1800144 (2018). https://doi.org/10.1002/aenm.201800144

[S10] M.H. Alfaruqi, V. Mathew, J. Song, S. Kim, S. Islam, Electrochemical zinc intercalation in lithium vanadium oxide: a high-capacity zinc-ion battery cathode. Chem. Mater. **29,** 1684-1694 (2017). https://doi.org/10.1021/acs.chemmater.6b05092

[S11] P. He, G. Zhang, X. Liao, M. Yan, X. Xu, Q. An, J. Liu, L. Mai, Sodium ion stabilized vanadium oxide nanowire cathode for high-performance zinc-ion batteries. Adv. Energy Mater. **8,** 1702463 (2018). https://doi.org/10.1002/aenm.201702463

[S12] B. Sambandam, V. Soundharrajan, S. Kim, M.H. Alfaruqi, J. Jo et al., Aqueous rechargeable zn-ion batteries: an imperishable and high-energy $Zn_2V_2O_7$ nanowire cathode through intercalation regulation. J. Mater. Chem. A **6,** 3850-3856 (2018). https://doi.org/10.1039/C7TA11237H

[S13] C. Xia, J. Guo, Y. Lei, H. Liang, C. Zhao, H.N. Alshareef, Rechargeable aqueous zinc-ion battery based on porous framework zinc pyrovanadate intercalation cathode. Adv. Mater. **30,** 1705580 (2018). https://doi.org/10.1002/adma.201705580

[S14] P. Hu, T. Zhu, X. Wang, X. Wei, M. Yan, Highly durable $Na_2V_6O_{16}\cdot 1.63H_2O$ nanowire cathode for aqueous zinc-ion battery. Nano Letters **18,** 1758-1763 (2018). https://doi.org/10.1021/acs.nanolett.7b04889

[S15] Y. Cai, F. Liu, Z. Luo, G. Fang, J. Zhou, A. Pan, S. Liang, Pilotaxitic $Na_{1.1}V_3O_{7.9}$ nanoribbons/graphene as high-performance sodium ion battery and aqueous zinc ion battery cathode. Energy Storage Mater. **13,** 168-174 (2018). https://doi.org/10.1016/j.ensm.2018.01.009

[S16] J.H. Jo, Y.K. Sun, S.T. Myung, Hollandite-type Al-doped $VO_{1.52}(OH)_{0.77}$ as a zinc ion insertion host material. J. Mater. Chem. A **5,** 8367-8375 (2017). https://doi.org/10.1039/C7TA01765K

[S17] G. Li, Z. Yang, Y. Jiang, C. Jin, W. Huang, X. Ding, Y. Huang, Towards polyvalent ion batteries: a zinc-ion battery based on NASICON structured $Na_3V_2(PO4)_3$. Nano Energy **25,** 211-217 (2016). https://doi.org/10.1016/j.nanoen.2016.04.051
